# Association of blood glucose level and hypertension in Elderly Chinese Subjects: a community based study

**DOI:** 10.1186/s12902-016-0119-5

**Published:** 2016-07-13

**Authors:** Qun Yan, Dongmei Sun, Xu Li, Guoliang Chen, Qinghu Zheng, Lun Li, Chenhong Gu, Bo Feng

**Affiliations:** Department of Endocrinology, Shanghai East Hospital, Tongji University School of Medicine, 150 Jimo Road, Shanghai, 200120 China; Puxing Community Health-Care Center, Pudong New District, 250 GuiChang Road, Shanghai, 200136 China

**Keywords:** Fasting plasma glucose, Diabetes, Hypertension, Cross-sectional study

## Abstract

**Background:**

There is a scarcity of epidemiological researches examining the relationship between blood pressure (BP) and glucose level among older adults. The objective of the current study was to investigate the association of high BP and glucose level in elderly Chinese.

**Methods:**

A cross-sectional study of a population of 2092 Chinese individuals aged over 65 years was conducted. Multiple logistic analysis was used to explore the association between hypertension and hyperglycemia. Independent risk factors for systolic and diastolic BP were analyzed using stepwise linear regression.

**Results:**

Subjects in impaired fasting glucose group (IFG) (*n* = 144) and diabetes (*n* = 346), as compared with normal fasting glucose (NFG) (*n* = 1277), had a significant higher risk for hypertension, with odds ratios (ORs) of 1.81 (95 % CI, 1.39–2.35) (*P* = 0.000) and 1.40 (95 % CI, 1.09–1.80) (*P* = 0.009), respectively. Higher fasting plasma glucose (FPG) levels in the normal range were still significantly associated with a higher prevalence of hypertension in both genders, with ORs of 1.24 (95 % CI, 0.85–1.80), *R*^2^ = 0.114, *P* = 0.023 in men and 1.61 (95 % CI, 1.12–2.30), *R*^2^ = 0.082, *P* = 0.010 in women, respectively, when compared with lower FPG. Linear regression analysis revealed FPG was an independent factor of systolic and diastolic BP.

**Conclusions:**

Our findings suggest that hyperglycemia as well as higher FPG within the normal range is associated with a higher prevalence of hypertension independent of other cardiovascular risk factors in elderly Chinese. Further studies are needed to explore the relationship between hyperglycemia and hypertension in a longitudinal setting.

## Background

Metabolic syndrome is defined as the occurrence of 3 of any of the 5 following factors: abdominal obesity, elevated triglyceride (TG), low high-density lipoprotein cholesterol (HDL-c), elevated blood pressure (BP) and elevated fasting glucose level [1]. It is well known that metabolic syndrome increases the risk for morbidity and mortality from cardiovascular diseases [2, 3]. Actually, the coexistence of hypertension and diabetes alone confers 2–3 times the risk for cardiovascular morbidity and mortality as for people without diabetes [4, 5]. Epidemiological research established that hypertension is more common in subjects with diabetes mellitus (DM) than in the general population [6, 7]. On the other hand, higher BP is associated with increased risk of diabetes [8–12], even in individuals with normotension [7]. However, current studies evaluating the relationship of BP with blood glucose level were limited to age of younger [8–10], or from across the age spectrum [6, 7, 13], did not separate out the data of elderly subjects. Furthermore, referral-based samples in most were mainly with hyperglycemia and hypertension [6, 7, 11, 12]. Some studies relied on self-reported DM or BP [8–10].

Considering the influence of hypertension or hyperglycemia on vascular disease and mortality are age-specific [13], we therefore conducted a cross-sectional survey study in a well-characterized community-based sample of individuals in East China aged over 65 to assess whether higher fasting plasma glucose (FPG) is independently associated with a higher prevalence of hypertension, and conversely, whether higher BP is associated with a higher prevalence of hyperglycemia in elder people.

## Methods

### Population

A cross-sectional study was conducted in Shanghai Pudong New Area between January 2012 and March 2012. Residents who had lived here over 5 years were recruited. Among the 2777 individuals who were aged over 65 year, 2250 subjects participated in the current study.

**Fig. 1:**
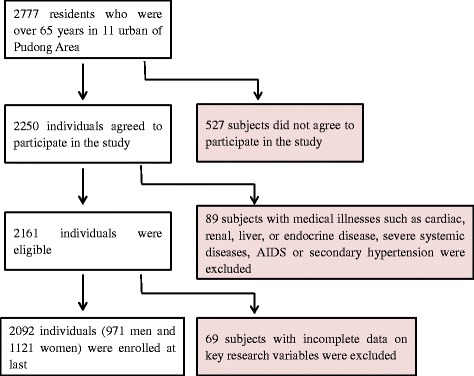
Study design and participant flow diagram for the present study

Inclusion criteria were: (i) men or women aged over 65 year; (ii) with no severe chronic disease or systemic disease; (iii) willing and able to give informed consent; and (iv) with complete data on key research variables. Subjects with medical diseases such as cardiac disease (heart failure), chronic liver and kidney dysfunction (including serum alanine aminotransferase > 120 IU/l, and aspartate aminotransaminase >80 IU/l, and serum creatinine > 2.0 mg/dl), acute infection, severe systemic diseases (e.g., cancer) or Acquired Immune Deficiency Syndrome (AIDS) as well as patients who had secondary hypertension were excluded (*n* = 89). Those with incomplete data on key research variables were also excluded (*n* = 69). Subsequently, 2092 individuals (971 men and 1121 women) were included in the current analysis (Fig. 1).

All participants were screened with regard to medical history (i.e., smoking, alcohol consumption, and medical treatments). Current smokers were defined as those who currently smoke every day or some days and who reported having smoked over 100 cigarettes during their lifetimes. Alcohol consumption was defined as having up to 20–30 g of ethanol per day for men and up to 10–20 g of ethanol per day for women. Details of this survey have been reported previously [14]. Written informed consent was obtained from all participants.

### BP Measurements

BP was measured using a mercury sphygmomanometer by an experienced physician. Subjects were in a seated position with an arm flexed at the level of the heart. After the cuff was wrapped around the upper arm with the cuff’s lower edge one inch above the antecubital fossa, the cuff was rapidly inflated to 180 mmHg. Then air was released from the cuff at a rate of 3 mm/s. During this period, the physician listened carefully with a stethoscope while simultaneously observing the sphygmomanometer. The first knocking sound (Korotkoff) was the subject’s systolic pressure. When the knocking sound disappeared, that was the diastolic pressure. BP was measured in either the right upper or left upper arm after each subject had been seated for at least 10 min. The arm with the higher blood pressure would be measured twice. The average was used for analysis. If the difference of SBP or DBP between the twice was over 5 mmHg, it would be measured for the third time, and then the average of three times was used for analysis.

Hypertension was defined as systolic blood pressure (SBP) ≥140 mmHg and/or diastolic blood pressure (DBP) ≥90 mmHg or reported receiving antihypertensive medication. Participants who were diagnosed hypertensive for the first time during this survey were called new-onset hypertension; participants who had a history of hypertension were called previously-diagnosed hypertension. High-normal BP was defined as not having hypertension, and with SBP 120–139 mmHg or DBP 80–90 mmHg; normal BP included SBP < 120 mmHg and DBP < 80 mmHg and with no antihypertensive treatment [15].

### DM Ascertainment

Participants were considered to have DM if fasting blood glucose ≥ 7.0 mmol/L or reported using insulin or oral hypoglycemic medication. Participants who were diagnosed DM for the first time during this survey were called new-onset DM, participants who had a history of DM were called previously-diagnosed DM. Normal fasting glucose (NFG) was defined as FPG < 6.1 mmol/L. Impaired fasting glucose (IFG) was defined as FPG ≥ 6.1 mmol/L but <7.0 mmol/L according to WHO criteria, 1999 [16].

### Covariates

Height and weight of all subjects were measured in light clothes with no shoes. Waist circumference was measured at the level midway between the lower rib margin and the iliac crest. Body mass index (BMI) was calculated as weight (kg) divided by the square of height (m).

Blood samples were collected from venous blood after overnight fasting, and FPG, total cholesterol and triglyceride were measured. Glucose measurement was done in a central lab in Shanghai East Hospital. Plasma fasting glucose level was determined in NaF preserved plasma using the glucose oxidase method by automatic analyzer, Accurizer Autochem 400 (Affinitech, China).

### Statistical analysis

Continuous data were summarized by the mean ± SD or median, and categorical data by number (percentages). All of the continuous data had skewed distribution even after logarithmically transformed. Mann–Whitney test (for data that was not normally distributed) and Chi-square test (for data that were categorical variables) were used to compare the differences between the Men and Women groups.

The participants were divided into 4 groups according to FPG levels: NFG (*n* = 1277), IFG (*n* = 144), New-onset DM (*n* = 325), and previously-diagnosed DM (*n* = 346). Binary logistic analysis was used to examine the association of hyperglycemia status with hypertension. Then NFG group was further divided into two subgroups according to the median FPG level by sex. Multiple logistic analyses were used to explore the associations between hypertension and hyperglycemia. Independent risk factors for systolic and diastolic pressure were analyzed using stepwise linear regression.

## Results

### Clinical characteristics

The baseline characteristics of the study population by sex were shown in Table 1. The study population consisted of 2092 (971 men and 1121 women, mean age 72.8 and 73.6 years, respectively). The prevalence of total DM in men and women were 23.1 % and 23.7 %, respectively (*P* = 0.381). The prevalence of hypertension in men and women were 45.3 % and 48.5 %, respectively (*P* = 0.077). There were no significant differences in the prevalence of IFG and high-normal BP between the sexes. Other metabolic and anthropometric variables, except for BMI and FPG, were significantly different between the sexes (all *P* < 0.05).

**Table 1:** Baseline characteristics by sex

Parameters	Men	Women	*P* value
Number	971	1121	
Age(y)	72.8 ± 6.4	73.6 ± 7.0	0.027
Current smoker n (%)	215(22.1 %)	27(2.4 %)	0.000
Alcohol consumption n (%)	143(14.7 %)	17(1.5 %)	0.000
T2DM n (%)	224(23.1 %)	266(23.7)	0.381
IFG n (%)	142(14.6 %)	167(14.9 %)	0.455
Hypertension n (%)	440(45.3 %)	544(48.5 %)	0.077
High-normal BP n (%)	427(44.0 %)	489(43.6 %)	0.453
BMI(kg/m^2^)	23.7 ± 3.3	23.9 ± 3.8	0.316
WC(cm)	87.9 ± 9.1	85.4 ± 10.3	0.000
SBP (mmHg)	133.7 ± 16.1	135.4 ± 15.8	0.013
DBP (mmHg)	78.6 ± 8.9	77.4 ± 8.6	0.002
FPG (mmol/L, Mean)	6.2 ± 1.6	6.3 ± 1.8	0.268
FPG (mmol/L, Median) ^a^	5.45	5.51	0.268
TG (mmol/L)	1.5 ± 0.9	1.6 ± 0.9	0.000
TC (mmol/L)	5.2 ± 1.0	5.8 ± 1.0	0.000

Categorical data were described as n (%) and continuous data were described as means ± SD

*BMI* body mass index, *WC* waist circumference, *SBP* systolic blood pressure, *DBP* diastolic blood pressure, *FPG* fasting plasma glucose, *TG* triglyceride, *TC* total cholesterol

^a^The median of FPG in subjects with normal fasting glucose level (<6.1 mmol/L)

*P* values < 0.05 were considered significant

### Association of FPG and risk of hypertension

After adjusting for sex, age, smoking, alcohol using, BMI, TG and TC levels, subjects in IFG and previously-diagnosed DM, as compared with NFG (<6.1 mmol/L), had a significant higher risk for hypertension in whole sample, with odds ratios (OR) of 1.81 (95 % CI, 1.39–2.35) (*P* = 0.000) and 1.40 (95 % CI, 1.09–1.80) (*P* = 0.009), respectively. Subjects in the IFG group were also associated with an increased OR of new-onset hypertension (OR 1.68 [95 % CI, 1.18–2.41], *P* = 0.004) when comparing with NFG. Of note, there were strong associations of hyperglycemic status with SBP and but not DBP (Table 2).

**Table 2:** Logistic regression analysis of association of hyperglycemia status with risk of hypertension^a^

	SBP ≥ 140 mmHg		DBP ≥ 90 mmHg		Total Hypertension		New-onset Hypertension	
	OR (95 % CI)	*P* value	OR (95 % CI)	*P* value	OR (95 % CI)	*P* value	OR (95 % CI)	*P* value
NFG	1	-	1	-	1	-	1	-
IFG	1.89(1.46–2.45)	0.000	1.31(0.94–1.84)	0.111	1.81(1.39–2.35)	0.000	1.68(1.18–2.41)	0.004
New-onset DM	1.44(0.99–1.65)	0.053	1.23(0.78–1.95)	0.375	1.35(0.93–1.94)	0.115	1.49(0.91–2.42)	0.113
Previously-diagnosed DM	1.46(1.13–1.87)	0.003	1.02(0.72–1.44)	0.913	1.40(1.09–1.80)	0.009	0.90(0.61–1.33)	0.592

*NFG* normal fasting glucose, *IFG* impaired fasting glucose, New-onset DM, newly diagnosed diabetes mellitus; Previously-Diagnosed DM, previously diagnosed diabetes mellitus; New-onset hypertension, newly diagnosed hypertension; OR (95 % CI), odds ratio (95 % confidence index); other abbreviations see Table 1

^a^Adjusted for sex, age, smoking, alcohol consumption, BMI, TG and TC level

*P* values < 0.05 were considered significant

The median of FPG was 5.45 mmol/L in men, and 5.51 mmol/L in women respectively in NFG group (Table 3). NFG group was further divided into two subgroups according to the median FPG level by sex. Even in the normal range of FPG, using below the median level of FPG as the reference, when adjusting for age, smoking and alcohol consumption (Model 1), higher FPG above the median was still significantly associated with a higher prevalence of hypertension in both gender, with OR of 1.93 (95 % CI, 1.60–2.34) (*R*^2^ = 0.028, *P* = 0.000) in men and 1.96 (95 % CI, 1.51–2.54) (*R*^2^ = 0.033, *P* = 0.000) in women respectively. After further adjusting for BMI, WC, TG and TC (Model 2), the association between higher FPG and the prevalence of hypertension was attenuated but remained statistically significant (OR 1.24 [95 % CI, 0.85–1.80], *R*^2^ = 0.114, *P* = 0.023 in men and 1.61 [95 % CI, 1.12–2.30], *R*^2^ = 0.082, *P* = 0.010 in women respectively). Similar associations were found between higher FPG levels and higher SBP in both genders. However, we did not find any association between higher FPG and higher DBP in both genders in Model 2.

**Table 3:** Logistic regression analysis of risk of hypertension in subjects with NFG (above vs. below the median fasting glucose level ^a^)

	Men			Women		
	OR (95 % CI, below vs above the median $)	*P* value#	Adjusted R^2^	OR (95 % CI, below vs above the median $)	*P* value^#^	Adjusted R^2^
Total Hypertension						
Model 1	1.93(1.60–2.34)	0.000	0.028	1.96(1.51–2.54)	0.000	0.033
Model 2	1.24(0.85–1.80)	0.023	0.114	1.61(1.12–2.30)	0.010	0.082
SBP ≥ 140 mmHg						
Model 1	2.04(1.54–2.70)	0.000	0.022	2.03(1.56–2.65)	0.000	0.026
Model 2	1.50(1.07–2.10)	0.019	0.058	1.27(0.93–1.74)	0.013	0.071
DBP ≥ 90 mmHg						
Model 1	1.21(0.84–1.75)	0.301	0.022	1.67(1.12–2.67)	0.011	0.063
Model 2	0.72(0.46–1.15)	0.167	0.048	1.35(0.85–2.12)	0.201	0.015

Abbreviations see Tables 1 and 2

Model 1, adjusted for age, smoking and alcohol using

Model 2, adjusted for age, smoking, alchol using, BMI, WC, TG and TC level

^a^The median fasting glucose level was 5.45 mmol/L in men and 5.51 mmol/L in women respectively

*P* values < 0.05 were considered significant

Table 4 showed the independent risk factors for SBP and DBP in whole sample and in individuals without hypoglycemic nor antihypertensive drugs (*n* = 924) by stepwise linear regression analysis. After adjusting for sex, age, smoking, alcohol consumption, BMI, WC, TC, and TG, FPG levels were significantly associated with SBP in both samples (β = 0.10, *R*^2^ = 0.013, *P* < 0.01 in whole sample and β = 0.12, *R*^2^ = 0.013, *P* < 0.01 in individuals without hypoglycemic or antihypertensive drugs respectively). As for DBP, FPG levels were also associated with DBP in individuals without hypoglycemic or antihypertensive drugs (β = 0.08, *R*^2^ = 0.006, *P* < 0.05).

**Table 4:** Stepwise linear regression analysis of risk factors for SBP and DBP

	All subjects (*n* = 2092)				Subjects without hypoglycemic or antihypertensive drugs (*n* = 924)			
	SBP		DBP		SBP		DBP	
	β	R^2^	β	R^2^	β	R^2^	β	R^2^
BMI (kg/m^2^)	0.15**	0.034	0.13**	0.032	0.15**	0.028	0.42	-
FPG (mmol/L)	0.10**	0.013	0.022	-	0.12**	0.013	0.08*	0.006
Age (years)	0.09**	0.008	-0.16**	0.019	0.07*	0.004	-0.17**	0.026
Smoking	−0.07	-	−0.03	-	−0.13*	0.012	−0.23	-
(yes/no)								
Alcohol consumption	0.06**	0.003	0.021	-	0.07*	0.004	0.026	-
(yes/no)								
TC (mmol/L)	0.05*	0.003	0.09**	0.005	0.04	-	0.061	-
TG (mmol/L)	0.07**	0.002	0.05*	0.007	0.01	-	-0.34	-
Male (vs.Female)	0.02	-	−0.09	-	0.03	-	0.109	-
WC(mmol/L)	0.57	-	0.61	-	0.09	-	0.14**	0.021

Abbreviations see Table 1

* *P* value < 0.05; **P value < 0.01

### Association of BP and risk of hyperglycemia

We explored the possible effect of high-normal BP and hypertension on the risk of IFG and DM by using multiple logistic regression analysis (Table 5). After adjusting for age, sex, smoking and alcohol consumption, when compared with the normal BP group, hypertensives had a significantly higher prevalence of IFG (OR 3.92 [95 % CI, 2.17–7.10], *P* < 0.01), and DM (2.83 [95 % CI, 1.83–4.38], *P* < 0.01) respectively, and subjects with high-normal BP had a similarly increased prevelance in IFG and DM (OR 2.07 [95 % CI, 1.13–3.78], *P* < 0.05, and OR 1.78 [95 % CI, 1.14–2.76], *P* < 0.05, respectively) (Table 5, Model 1). When further adjusting for BMI, WC, TG, and TC, the associations between hypertension and IFG and DM remained statistically significant, though attenuated (OR 3.17 [95 % CI, 1.70–5.68], *P* < 0.01, and 1.93 [95 % CI, 1.22–3.04], *P* < 0.01, respectively). Similar associations were also seen between high-normal BP and IFG, but not DM (Table 5, Model 2).

**Table 5:** Multiple logistic regression analysis of risk of IFG and DM

	IFG		DM	
	OR (95 % CI)	Adjusted R^2^	OR (95 % CI)	Adjusted R^2^
Model 1 ^a^		0.045		0.028
Age (years)	1.01(0.99–1.03)		1.00(0.99–1.02)	
Male (vs. female)	0.95(0.73–1.25)		0.99(0.78–1.24)	
Smoking(yes/no)	0.92(0.68–1.08)		1.01(0.85–1.21)	
Alcohol consumption (yes/no)	1.06(0.84–1.43)		0.93(0.76–1.41)	
High-normal BP	2.07(1.13–3.78)*		1.78(1.14–2.76)*	
(vs. normal BP)				
Hypertension	3.92(2.17–7.10)**		2.83(1.83–4.38)**	
(vs. normal BP)				
Model 2 ^b^		0.095		0.140
Age (years)	1.01(0.99–1.03)		1.01(0.99–1.03)	
Male (vs. female)	0.96(0.71–1.30)		0.97(0.81–1.18)	
Smoking(yes/no)	0.83(0.65–1.05)		0.92(0.74–1.14)	
Alcohol consumption (yes/no)	1.09(0.86–1.39)		0.99(0.77–1.28)	
BMI (kg/m^2^)	1.02(0.95–1.09)		1.04(0.99–1.10)**	
WC(mmol/L)	1.03(1.00–1.06)*		1.04(1.02–1.06)**	
TG (mmol/L)	1.42(1.22–1.66)**		1.55(1.36–1.77)**	
TC (mmol/L)	0.96(0.84–1.10)		0.95(0.85–1.06)	
High-normal BP	1.87(1.03–3.44)*		1.46(0.94–2.31)	
(vs. normal BP)				
Hypertension	3.17(1.70–5.68)**		1.93(1.22–3.04)**	
(vs. normal BP)				

Abbreviations see Tables 1 and 2

Normal BP, SBP < 120 mmHg and DBP < 80 mmHg and not using antihypertensive medication; high-normal BP, SBP 120–139 mmHg or DBP 80–90 mmHg; hypertension, SBP ≥ 140 mmHg and/or DBP ≥ 90 mmHg or using antihypertensive medication

^a^Model 1: Logistic regression analysis with IFG and DM as dependent variable respectively, and age, male (vs. female), smoking, alcohol consumption, and high-normal BP (vs. normal BP) and hypertension (vs. normal BP) as independent variables

^b^Model 2: Model 1 + BMI, WC, TG and TC

* *P* value < 0.05; **P value < 0.01

## Discussion

The close relationship between diabetes and hypertension has been recognized since the 1980s [6, 10], but how much this effect varies by race-ethnicity, age or levels of traditional risk factors is uncertain. The extent to which glucose concentration is associated with elevated blood pressure is also uncertain. The data for pure elders in China is more limited. In our present study, we aimed to explore the association of diabetes and FPG concentration with hypertension or high-normal BP in a select groups of elderly subjects in China. We found that, compared with subjects with NFG, subjects with IFG and previously-diagnosed DM had a higher prevalence of hypertension. Even among the subjects with NFG, higher FPG was still associated with a higher prevalence of hypertension after adjusting for covariates such as age, smoking, alcohol consumption, BMI, WC, TG, and TC level. FPG was an independent factor of systolic and diastolic BP. Conversely, multiple analyses revealed that subjects with hypertension also had a higher prevalence of IFG and DM.

Our study is comply with other studies that have indicated an increased risk for hypertension and other cardiovascular risk factors in diabetic individuals as well as in impaired glucose regulation than those with NFG [6, 7, 17, 18]. Our study extends this observation by illustrating that a high but normal FPG level was also a risk factor for hypertension in elderly Chinese subjects, and this relation was independent of other traditional cardiovascular risk factors. What’s more, we found that elevated FPG was independently associated with an increased risk of SBP and DBP. This is also in line with two recent studies [19, 20]. In Di Bonito P’s study among Southern European Caucasian children and adolescents (age 6–16 years), they found that subjects with high-normal FPG (83–99 mg/dL) had 1.57–2.23 times the risk for hypertension when compared with subjects with low-normal FPG (≤82 mg/dL) independent of BMI [19]. In a cohort study of a Japanese population (age 20–80 years), Y Heianza found that elevated concentration of FPG in normal range (<4.9 mmol/L vs. 4.9–5.6 mmol/L) was associated with higher risk of developing hypertension (OR 1.35–1.39) during a 5-year follow-up after adjusting for age and gender [20].

Our data also matches with other epidemiological data demonstrating that basal BP and BP progression are strong and independent predictors of incident hyperglycemia [7, 10–12, 18], and is also consistent with clinical trial data demonstrating that lower BP are associated with lower risk of cardiovascular diseases [13]. In the Women’s Health Study in the USA by David Conen et al, a baseline BP of 130-139/85-89 mmHg and hypertension will increase risk of incident Type 2 diabetes in the following 10 years by 1.45–2.03 times [7]. In a recent study of Chinese subjects in Hong Kong aged over 18 years, Sau NF found that subjects with IFG were more likely to develop diabetes within 5 years if they were hypertensive (aHR = 1.44) [11]. A large Japanese cohort study also showed that having both high-normal BP and hypertension in middle age was associated with increased risk of developing diabetes [9]. In our present study, we also found high-normal BP associated with risk of IFG after adjusting for conventional risk factors.

Although the association between hypertension and hyperglycemia has long been discussed, the underlying biological basis mediating this clinical association remains unclear. Hyperinsulinemia, an important component of the metabolic syndrome, may be one of the main factors [21]. Insulin resistance, which further contributes to hyperinsulinemia, often progresses to Type 2 diabetes [22]. In addition, the resistance in the hemodynamic properties of insulin may impair the blood flow in peripheral tissues, which indirectly contributes to the development of the atherogenic dysglycemia and dyslipidemia [23]. Hyperinsulinemia may directly contribute to elevation of blood pressure by increasing renal sodium retention [24] and enhancing sympathetic nervous system activity [25], whereas chronic sympathetic nervous system overactivity may contribute to a further increase of insulin resistance creating a vicious circle that may lead to the development of hypertension and diabetes. In addition, insulin resistance is also associated with a decreased vasodilatory response to insulin in peripheral tissue [26, 27] and an increased vasoconstrictor response to various vasopressors [28, 29], which mainly results the increase of systolic blood pressure. The pathophysiological mechanism linking insulin and hypertension is complex and still needs to be fully elucidated.

Our present study adds to the literature by demonstrating that hyperglycemia is closely correlated with hypertension in a population of the elderly. We were able to explore the associations between blood glucose levels and BP in a relative large, well-defined cohort. However, several potential limitations of the study merit discussion. First, because of the observational nature of the study, we cannot infer causality from our present data. Second, lack of evaluation of postprandial blood glucose level and repeated FPG measurement should underestimate the prevalence of diabetes in this population. As this was a survey based on the elderly population, postprandial blood glucose measurement may pose certain problems for the elderly, as they would need to wait 2 h for venous draw blood after a meal. Third, although multiple potential confounders have been adjusted for, we cannot completely rule out the residual confounders attributed to the inaccurate measurement of confounders, or some unmeasured confounders.

## Conclusions

Our study suggests that hyperglycemia as well as a high but normal FPG level was associated with a higher prevalence of hypertension independent of other cardiovascular risk factors among elderly Chinese. On the other hand, hypertension, even high-normal BP, was also associated with a higher prevalence of hyperglycemia. Further studies are needed to explore the relationship between hyperglycemia and hypertension in a longitudinal setting.

## Abbreviations

BP, blood pressure; TG, triglyceride; HDL-c, high-density lipoprotein cholesterol; DM, diabetes mellitus; FPG, fasting plasma glucose; AIDS, acquired immune deficiency syndrome; SBP, systolic blood pressure; DBP, diastolic blood pressure; NFG, normal fasting glucose; IFG, impaired fasting glucose; BMI, body mass index; TC, total cholesterol
